# Methionine Deprivation Reveals the Pivotal Roles of Cell Cycle Progression in Ferroptosis That Is Induced by Cysteine Starvation

**DOI:** 10.3390/cells11101603

**Published:** 2022-05-10

**Authors:** Takujiro Homma, Sho Kobayashi, Junichi Fujii

**Affiliations:** 1 Department of Biochemistry and Molecular Biology, Graduate School of Medical Science, Yamagata University, Yamagata 990-9585, Japan; jfujii@med.id.yamagata-u.ac.jp; 2 Department of Food, Life and Environmental Science, Faculty of Agriculture, Yamagata University, Yamagata 990-9585, Japan; skobayashi@tds1.tr.yamagata-u.ac.jp

**Keywords:** ferroptosis, glutathione, cell cycle, methionine, cysteine

## Abstract

Ferroptosis, a type of iron-dependent necrotic cell death, is triggered by the accumulation of excessive lipid peroxides in cells. Glutathione (GSH), a tripeptide redox molecule that contains a cysteine (Cys) unit in the center, plays a pivotal role in protection against ferroptosis. When the transsulfuration pathway is activated, the sulfur atom of methionine (Met) is utilized to generate Cys, which can then suppress Cys-starvation-induced ferroptosis. In the current study, we cultured HeLa cells in Met- and/or cystine (an oxidized Cys dimer)- deprived medium and investigated the roles of Met in ferroptosis execution. The results indicate that, in the absence of cystine or Met, ferroptosis or cell cycle arrest, respectively, occurred. Contrary to our expectations, however, the simultaneous deprivation of both Met and cystine failed to induce ferroptosis, although the intracellular levels of Cys and GSH were maintained at low levels. Supplementation with S-adenosylmethionine (SAM), a methyl group donor that is produced during the metabolism of Met, caused the cell cycle progression to resume and lipid peroxidation and the subsequent induction of ferroptosis was also restored under conditions of Met/cystine double deprivation. DNA methylation appeared to be involved in the resumption in the SAM-mediated cell cycle because its downstream metabolite S-adenosylhomocysteine failed to cause either cell cycle progression or ferroptosis to be induced. Taken together, our results suggest that elevated lipid peroxidation products that are produced during cell cycle progression are involved in the execution of ferroptosis under conditions of Cys starvation.

## 1. Introduction

Ferroptosis is a newly characterized form of cell death that is caused by the accumulation of intolerable levels of lipid peroxides in the cell via an iron-mediated Fenton reaction [1,2]. The resulting lipid peroxides that are generated in membrane phospholipids cause ferroptotic cell death by disturbing the integrity of the plasma membrane. Glutathione (GSH), a tripeptide redox molecule that contains a cysteine (Cys) unit in the center, plays a pivotal role in protection against ferroptosis. GSH serves as an electron donor to glutathione peroxidase 4 (GPX4), which is a protective enzyme that potently protects against ferroptosis by reductively detoxifying lipid peroxides [3,4]. Because intracellular Cys levels are maintained at relatively low levels, the other two amino acids are relatively abundant. It therefore follows that the availability of Cys tends to restrict GSH synthesis. xCT, the core transporter protein of system x_c_^−^, is responsible for the cellular uptake of cystine, a dimeric form of oxidized Cys in which the two units are linked by a disulfide bridge [5]. Ferroptosis is typically induced under conditions of an insufficient supply of Cys, such as cystine deprivation in the culture medium and the inhibition of xCT by erastin, a compound that disrupts the uptake of cystine from the extracellular milieu. A Cys insufficiency results in a decrease in GSH synthesis, leading to elevated levels of lipid peroxidation products due to GPX4 dysfunction and the subsequent destruction of membranes.

Cys is metabolically produced by the transsulfuration pathway in conjunction with methionine (Met) metabolism and may fulfil the requirement in some organs under normal physiological conditions [6]. In addition to protein synthesis, Met is the precursor for S-adenosylmethionine (SAM), which donates a methyl group to several acceptor molecules such as DNA, RNA, proteins, and phospholipids. The resulting demethylated molecule, S-adenosylhomocysteine (SAH), yields homocysteine (Hcy) which may enter the remethylation pathway to produce Met through accepting a methyl group from other donor compounds. In the synthesis of Cys, however, Hcy binds serine to cystathionine (Cysta) by the catalytic action of cystathionine *β*-synthase (CBS) and is subsequently cleaved by cystathionine *γ*-lyase (CSE) to give Cys. Thus, the transsulfuration pathway actively supplies Cys to cells in some organs, notably the liver, where it appears to allow xCT-deficient primary hepatocytes to survive for several days in the cystine-free medium [7]. The significance of the supply of Cys via the transsulfuration pathway is supported either by the knockout of the corresponding genes or the inhibition of the transsulfuration pathway by propargylglycine, an inhibitor of CSE [8]. Some cancer cells express higher levels of transsulfuration pathway enzymes, such as CBS and CSE, compared to normal cells [9,10], which may render them resistant against oxidative damage caused by chemotherapy or radiation therapy. In fact, in cases where the supply of extracellular Cys is limited, transsulfuration-mediated Cys synthesis is a metabolic pathway in cancer cells that is critical for their survival [11]. Cell growth is arrested in Met-free medium, which characteristically occurs in cancer cells [12,13,14]. Because Met is an essential amino acid, a defect in Met supply could impair the protein synthesis required for cell cycle progression. The inhibition of protein synthesis during the G2 phase causes a delay in mitotic progression, but may simultaneously preserve Cys for GSH synthesis, thus making cells resistant to oxidative insult [15]. Another possibility is that the cell cycle is regulated by Met metabolites per se. During DNA replication at the S-phase of the cell cycle, methylation at the 5-position of cytosine needs to be maintained in daughter DNA by DNA methyltransferase DNMT1 [16]. A loss of DNA methylation by means of DNMT1 gene ablation causes hemimethylation in the CpG island and leads to cell cycle arrest at the G2 phase and eventual death [17]. Thus, the cell cycle arrest observed in the Met-deficient cultures could, at least partly, be explained by an insufficient supply of SAM from Met metabolism. Indeed, restricting dietary Met reportedly suppresses the proliferation and progression of a variety of tumors [18,19,20], although the mechanism responsible for this is still under debate. Therefore, a detailed understanding of the metabolic flow of the Cys-GSH axis is needed before therapeutic approaches can be developed for the treatment of multiple types of cancer [21,22].

Despite the significant roles of Met in Cys metabolism and cancer biology, the interplay between Met metabolism and ferroptosis in cancer cells has not been fully investigated. We had speculated that a combined deprivation of Met and cystine induces ferroptosis more effectively in vitro. Contrary to these predictions, however, we found that cystine deprivation alone induced ferroptosis while Met/cystine double deprivation remarkably suppressed ferroptosis, leading to the cell survival in HeLa cells. Moreover, our results also suggest that cell cycle repression under this condition may be involved in the resistance to ferroptosis.

## 2. Materials and Methods

### 2.1. Cell Cultures and Chemicals

HeLa cells, a human cervical carcinoma cell line, were obtained from the American Type Culture Collection (ATCC, Manassas, VA, USA). HeLa/Fucci cells, a subline of the HeLa cell line that expresses a cell cycle marker Fucci [23], and Hepa 1–6 cells, a mouse hepatoma-derived cell line, were obtained from the RIKEN Bioresource Center (Tsukuba, Japan). All of the cell types were maintained in Dulbecco’s Modified Eagle’s Medium (DMEM; Fujifilm Wako Pure Chemical, Osaka, Japan; 044-29765) supplemented with 10% fetal bovine serum (FBS; Biowest, Riverside, MO, USA) and a penicillin-streptomycin solution (Fujifilm Wako Pure Chemical; 168-23191) at 37 °C in a 5% CO_2_ incubator. Medium that was free of both Met and cystine was prepared by using DMEM/high glucose/no glutamine/ no methionine/no cystine (Thermo Fisher Scientific, Waltham, MA, USA; 21013-024) supplemented with 10% FBS, a penicillin-streptomycin solution, 4 mM L-glutamine (Fujifilm Wako Pure Chemical), and 1 mM sodium pyruvate (Fujifilm Wako Pure Chemical). For cystine deprivation, the above medium was further supplemented with 0.2 mM L-methionine (PEPTIDE INSTITUTE, Osaka, Japan). Erastin (Item No. 17754), SAM (Item No. 16376), SAH (Item No. 13603), and Hcy (Item No. 30852) were purchased from Cayman Chemical (Ann Arbor, MI, USA).

### 2.2. Evaluation of Cytotoxicity of Cells

Cells were seeded at an initial density of (1.0 × 10^5^/mL). Cytotoxicity was determined by means of a lactate dehydrogenase (LDH) assay as described previously [24]. The reaction mixture contained 20 μL of culture medium, 0.3 mM NADH, 1 mM sodium pyruvate, and 200 mM sodium phosphate buffer, at pH 7.4 in a total volume of 100 μL. Initial activities were calculated from the rate of disappearance of NADH during the starting linear phase of the reaction by monitoring the absorbance at 340 nm.

### 2.3. Hoechst and Propidium Iodide (PI) Double Staining

Cells were incubated with Hoechst 33342 and PI in the medium (2 μg/mL each) for 20 min at 37 °C in a 5% CO_2_ incubator. The cells were then washed, and images were obtained using a BZ-X700 microscope (KEYENCE, Osaka, Japan).

### 2.4. Liquid Chromatography-Mass Spectrometry (LC-MS) Analyses

LC-MS analyses of the intracellular contents of Met-Cys-related metabolites were performed as described previously [24]. System control, data acquisition, and quantitative analysis involved the use of the Xcalibur software program v2.2. https://www.thermofisher.com/order/catalog/product/OPTON-30965 (accessed on 9 March 2022)(Thermo Fisher Scientific). Standard curves for amino acids, GSH-NEM, and Cys-NEM showed linearity in the concentration ranges examined.

### 2.5. Flow Cytometry

For the detection of lipid peroxidation products, HeLa cells were incubated with 10 μM C11-BODIPY^581/591^ (Thermo Fisher Scientific) for 30 min following the manufacturer’s instructions and then washed with PBS. After trypsinization, the cells were collected and subjected to flow cytometry (FACSCanto™ II, BD Biosciences, Tokyo, Japan). For an analysis of the cell cycle, HeLa cells were fixed in ice-cold 70% ethanol, and stored at −20 °C until used. The fixed cells were recovered by centrifugation and then resuspended in a staining solution containing 1 mg/mL RNaseA and 100 μg/mL PI in PBS at room temperature for 20 min. The stained cells were subjected to flow cytometry. Alternatively, for the real-time analysis of the cell cycle, HeLa/Fucci cells [23] were trypsinized and subjected to flow cytometry without fixation.

### 2.6. Detection of Intracellular Ferrous Iron under Fluorescent Microscopy

Cells were incubated with 5 μM FeRhoNox™-1 (Goryo Chemical, Sapporo, Japan) in the culture medium for 1 h according to the manufacturer’s instructions. The cells were then washed with PBS and images were obtained using a BZ-X700 microscope.

### 2.7. Immunostaining

Cells were fixed in 4% formaldehyde for 30 min at room temperature. After washing twice with PBS, the cells were permeabilized for 5 min with 0.5 % Triton X-100 in PBS and treated with 2N HCl for 30 min at room temperature. Subsequently, the cells were blocked with PBS containing 1% BSA and stained with an anti-5-Methylcytosine mouse mAb (Merck Millipore, Burlington, MA, USA; 162 33 D3) diluted 1:500 in blocking solution, followed by a rabbit anti-mouse IgG (H+L) Alexa Fluor^®^ 488 conjugate antibody (Thermo Fisher Scientific, dilution 1:500) for 60 min at room temperature. All images were acquired using a BZ-X700 microscope (KEYENCE).

### 2.8. Statistical Analysis

Statistical analyses were performed using the GraphPad Prism version 6.0 for Mac (accessed on 9 March 2022) (San Diego, CA, USA). A *p*-value of less than 0.05 was considered to be significant.

## 3. Results

### 3.1. Methionine Deprivation Suppresses Ferroptosis Induced by Cystine Deprivation

**Figure 1:**
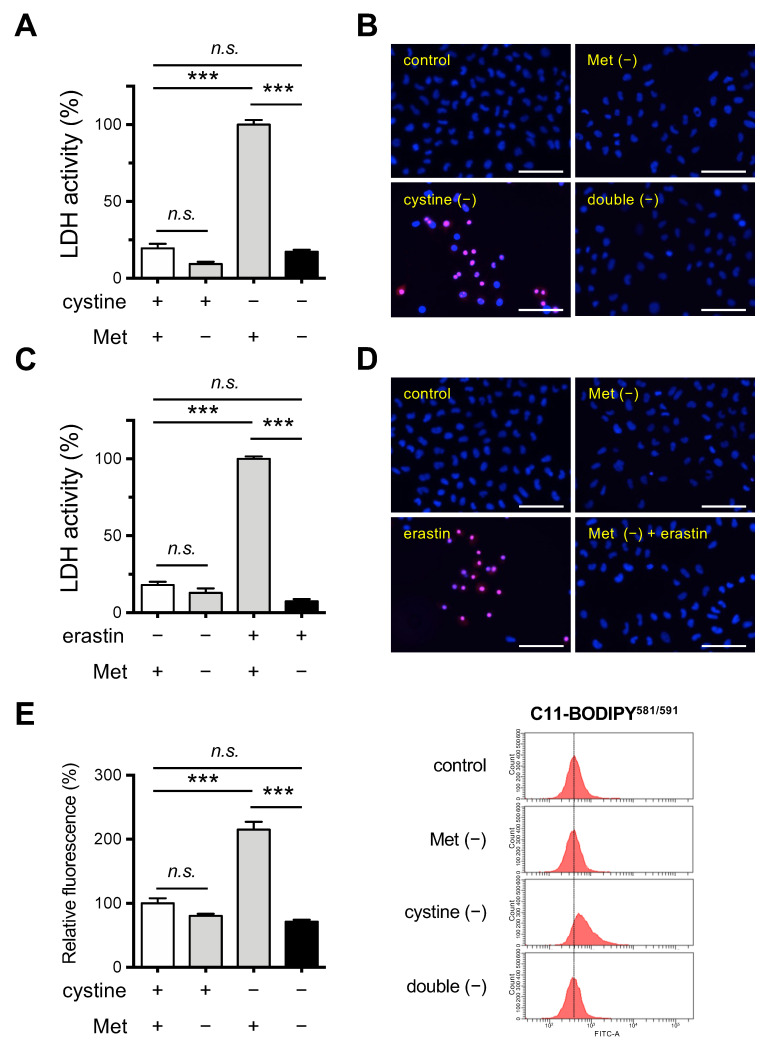
Effects of Met/cystine double deprivation on the induction of ferroptosis in HeLa cells. (**A**) Cytotoxicity of cells assessed by measuring released LDH activity. HeLa cells were incubated in complete (control), Met-free, cystine-free, or Met/cystine double-free medium for 24 h. Data represent the mean ± SEM (*n* = 3). ***: *p* < 0.001 (Tukey’s test). n.s.: not significant. (**B**) Plasma membrane integrity of cells that had been treated under the same conditions as (**A**) was assessed by PI staining. The cells were stained with PI (red) and Hoechst 33342 (blue). Bars: 100 µm. (**C**) Cytotoxicity of cells assessed by measuring released LDH activity. HeLa cells were incubated in complete (control) or Met-free medium in the presence or absence of 10 μM erastin for 24 h. Data represent the mean ± SEM (*n* = 3). ***: *p* < 0.001 (Tukey’s test). n.s.: not significant. (**D**) Plasma membrane integrity of cells that had been treated under the same conditions as (**C**) was assessed by PI staining. The cells were stained with PI (red) and Hoechst 33342 (blue). Bars: 100 µm. (**E**) Lipid peroxide production assessed by flow cytometry using C11-BODIPY^581/591^. HeLa cells were incubated in complete (control), Met-free, cystine-free, or Met/cystine double-free for 18 h, treated with 10 μM C11-BODIPY^581/591^, and then subjected to flow cytometry. Values for the fluorescence relative to cells cultured in control medium are shown (*n* = 3). Data represent the mean ± SEM. ***: *p* < 0.001 (Tukey’s test). n.s.: not significant.

To assess the influence of the co-deficiency of Met in ferroptosis that is induced by cystine deprivation, we cultivated Hela cells in Cys- and/or Met-free medium. Cytotoxicity of the HeLa cells was assessed by measuring the released LDH activity and PI staining. The results indicated that necrotic death was apparent in cells that were cultured in the absence of cystine, which was consistent with our previous observation in mouse Hepa 1–6 cells [24,25], but no necrotic death was observed in the absence of Met or in the absence of both Met and cystine (Figure 1A,B). Thus, cystine deprivation alone induced cell death, but the co-deprivation of Met suppressed cell death somewhat, at least for the 24 h culture period. 

We also inhibited xCT in cells that were cultured in complete and Met-free medium by treating them with erastin. The erastin treatment induced cell death in HeLa cells, consistent with the conclusion that an insufficient Cys supply was responsible for the cell death caused by the inhibition of xCT (Figure 1C,D). In addition, the cytotoxic effect of erastin was completely blocked in cells that were cultured in Met-free medium. We next examined the issue of whether lipid peroxidation was augmented in these cells using a lipid peroxide-sensitive fluorescent probe C11-BODIPY^581/591^. The fluorescent intensity was elevated in cells that were cultured in cystine-free medium while Met deprivation failed to induce detectable lipid peroxide production (Figure 1E). Consistent with the results for the cell death assays, the increased fluorescent intensity was suppressed when HeLa cells were cultured under Met/cystine double-free conditions (Figure 1E). In order to confirm that this phenomenon was independent of cell type, we investigated the effect of Met/cystine double deprivation on mouse hepatoma-derived Hepa 1–6 cells in which ferroptosis had been previously implicated [24]. Consistent with the results for HeLa cells, Met/cystine double-free conditions did not induce ferroptosis in Hepa 1–6 cells while cystine deprivation alone robustly induced ferroptosis, as evidenced by LDH assays (Supplementary Figure S1A), and PI staining (Supplementary Figure S1B). Similar results were obtained in the case of a combination of erastin treatment and Met deprivation in Hepa 1–6 cells (Supplementary Figure S1C,D). An increase in the fluorescence intensity of the lipid peroxidation probe C11-BODIPY^581/591^ was also observed under conditions of cystine deprivation in Hepa 1–6 cells, and again this increase was suppressed by co-deprivation with Met (Supplementary Figure S1E). Collectively, these results suggest that Met deprivation suppresses the ferroptosis that is induced by Cys starvation in a cell-type independent manner.

**Figure 2:**
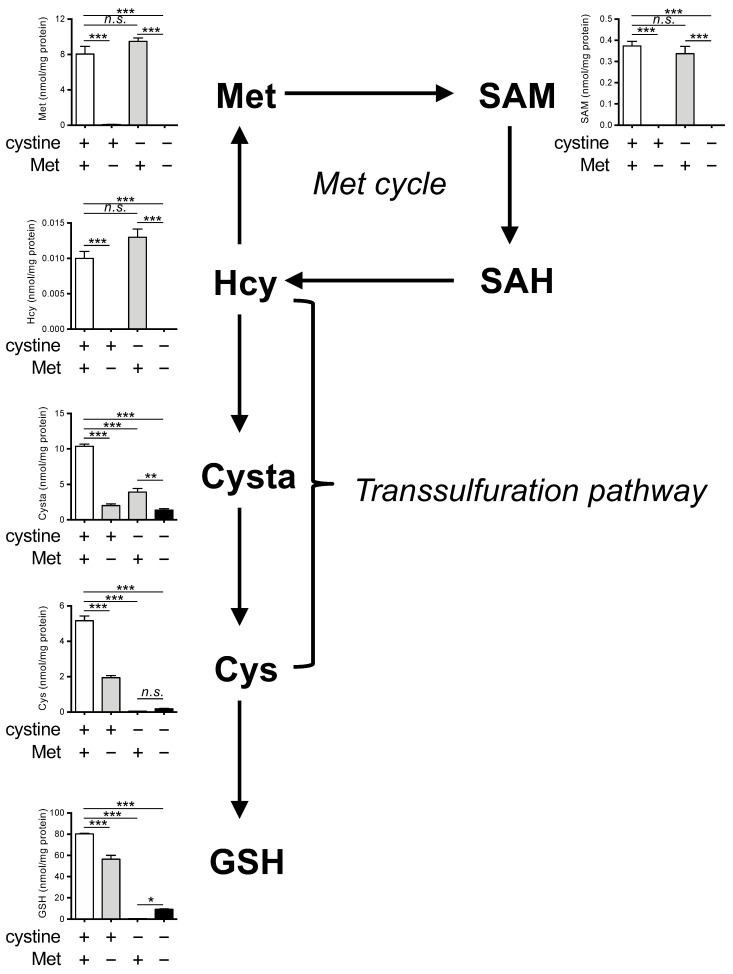
Effects of Met/cystine double deprivation on Met-cycle metabolites. Intracellular concentration of Met, SAM, SAH, homocysteine (Hcy), cystathionine (Cysta), Cys and GSH in HeLa cells 24 h after culture in complete (control), Met-free, cystine-free, or Met/cystine double-free medium. The SAH levels were below detectable levels in our assay method. Data are presented as the mean ± SEM (*n* = 3). *: *p* < 0.05, **: *p* < 0.01, ***: *p* < 0.001 (Tukey’s test). n.s.: not significant.

We next determined the intracellular levels of Met and its metabolites (SAM, SAH, Hcy, cystathionine (Cysta), Cys, and GSH) in HeLa cells cultured in complete, Met-free, cystine-free, or double-free conditions. We observed a significant decrease in the levels of Met, SAM, and Hcy under Met-free and double-free conditions (Figure 2). The SAH levels were below the limits of detection in our assay method and are not presented. The levels of Cysta were also significantly decreased in Met-free, cystine-free, and double-free conditions. Both Cys and GSH were completely absent in cystine-free and double-free conditions while the GSH levels were slightly restored in the case of double-free conditions. Collectively, these results indicate that the intracellular levels of Cys and GSH are irrelevant in the suppression of ferroptosis under double-free conditions.

### 3.2. Supplementation of SAM Restores Ferroptosis Induction under Met/Cystine Double-Free Conditions

**Figure 3:**
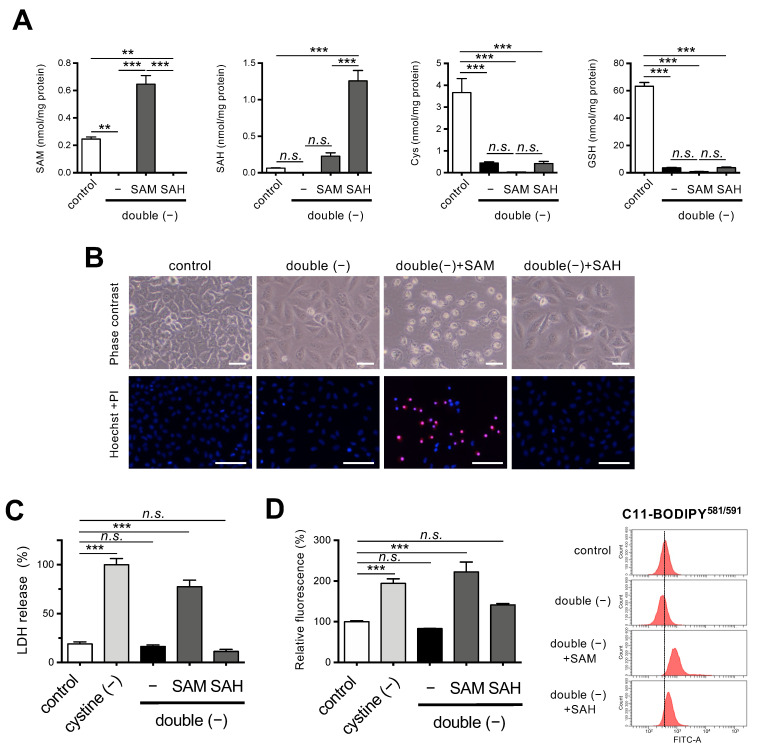
Effects of SAM on ferroptosis under Met/cystine double-free conditions. (**A**) Intracellular concentration of SAM, SAH, Cys, and GSH in HeLa cells 24 h after culturing in complete (control) or Met/cystine double-free medium supplemented with or without 0.2 mM SAM or 0.2 mM SAH. Data are presented as the mean ± SEM (*n* = 3). (**B**) Top, representative phase-contrast images of cells that had been treated under the same conditions as (**A**). Note that SAM supplementation increased collapsed ferroptotic cells with plasma membrane rupture. Bottom, plasma membrane integrity of cells was assessed by PI staining. HeLa cells were incubated in complete (control) or Met/cystine double-free medium supplemented with or without 0.2 mM SAM or 0.2 mM SAH for 24 h. The cells were stained with PI (red) and Hoechst 33342 (blue). Bars: 100 µm. (**C**) Cytotoxicity of cells assessed by measuring released LDH activity. HeLa cells were incubated in complete (control), cystine-free, or Met/cystine double-free medium supplemented with or without 0.2 mM SAM or 0.2 mM SAH for 24 h. Data represent the mean ± SEM (*n* = 3). (**D**) Lipid peroxide production assessed by flow cytometry using C11-BODIPY^581/591^. HeLa cells were incubated in complete (control), cystine-free, or Met/cystine double-free medium supplemented with or without 0.2 mM SAM or 0.2 mM SAH for 18 h, treated with 10 μM C11-BODIPY^581/591^, and then subjected to flow cytometry. Values for the fluorescence relative to cells cultured in control medium are shown (*n* = 3). **: *p* < 0.01, ***: *p* < 0.001 (Tukey’s test). n.s.: not significant.

To examine the role of Met metabolism in the suppression of ferroptosis under double-free conditions, we cultured HeLa cells in double-free medium supplemented with SAM or SAH. SAM supplementation resulted in increased intracellular levels of SAM under double-free conditions (Figure 3A). SAH supplementation effectively increased intracellular levels of SAH compared with SAM supplementation whereas both SAM and SAH supplementation did not restore intracellular levels of Cys and GSH (Figure 3A).

Supplementation with SAM but not SAH restored the sensitivity of HeLa cells to ferroptosis under double-free conditions, as evidenced by an increase in collapsed ferroptotic cells with ruptured plasma membranes with SAM supplementation (Figure 3B). These results were also confirmed by PI staining and LDH assays (Figure 3B,C). As expected, the fluorescent intensity of C11-BODIPY^581/591^ was elevated in the cells that were grown in media supplemented with SAM but not SAH, suggesting that SAM supplementation induced lipid peroxidation even under double-free conditions (Figure 3D). The cell death following the SAM supplementation was robustly rescued by ferrostatin-1 (Supplementary Figure S2A,B), a specific inhibitor of ferroptosis [1]. Since the accumulation of free ferrous iron is one of the hallmarks of ferroptosis [1,2], we explored the mobilization of iron in the cells by means of a ferrous iron-specific fluorescent probe FeRhoNox™-1 [26], and the results showed that SAM supplementation increased the fluorescent intensity of the cells to some extent (Supplementary Figure S2C). These collective results suggest that ferroptosis is involved in the cell death following SAM supplementation.

### 3.3. Supplementation of SAM in a Double-Depleted Environment Induces Ferroptosis as well as Releasing Cell Cycle Arrest in HeLa Cells

**Figure 4:**
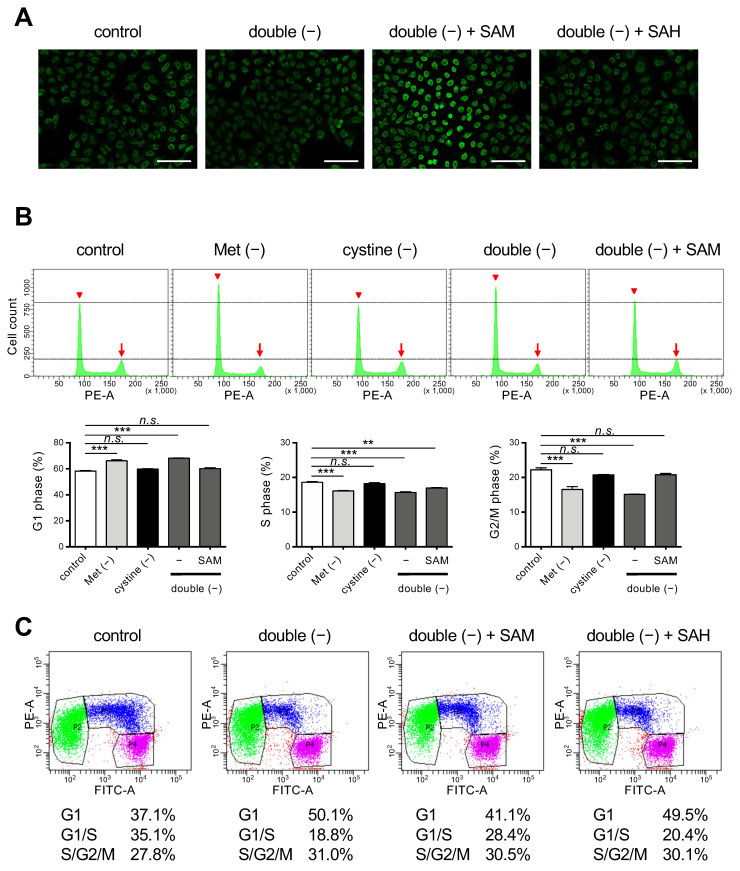
Effects of SAM on cell cycle regulation under Met/cystine double-free conditions. (**A**) Immunostaining with anti-5mC antibody. HeLa cells were incubated in complete (control) or Met/cystine double-free medium supplemented with or without 0.2 mM SAM or 0.2 mM SAH for 12 h, and then stained with an anti-5mC antibody (green). Bars: 100 µm. (**B**) Cell cycle analyses by PI staining. HeLa cells were incubated in complete (control), Met-free, cystine-free, or Met/cystine double-free medium supplemented with or without 0.2 mM SAM for 18 h. After staining with PI, the HeLa cells were analyzed by flow cytometry. Arrowheads and arrows indicate the positions of 2N and 4N, respectively. Bottom panels indicate the distribution of the cell-cycle phases. Data represent the mean ± SEM (*n* = 3). **: *p* < 0.01, ***: *p* < 0.001 (Dunnett’s test). n.s.: not significant. (**C**) Cell cycle analysis with the Fucci indicators. HeLa/Fucci cells were incubated in complete (control) or Met/cystine double-free medium supplemented with or without 0.2 mM SAM or 0.2 mM SAH for 18 h, and then subjected to flow cytometry. Cell populations in G1-, G1-/S-, and S-/G2-/M-phase are represented by green, blue, and purple, respectively. Values indicate the proportion of cells in each phase of the cell cycle (the mean of triplicate cultures).

Because SAH lacks the methyl group that is present in SAM, our results suggest the importance of the donation of the methyl group for the execution of ferroptosis in cystine deprivation. A methyl group from SAM is transferred to the 5-position of cytosine residues in DNA, resulting in methylated cytosine referred to as 5mC, which then modulates cell cycle progression and epigenetic modification [27]. We confirmed that supplementation with SAM but not SAH caused an increase in intracellular 5mC levels under double-free conditions (Figure 4A). We next examined the issue of whether the cell cycle was affected. Flow cytometry of HeLa cells that had been stained with PI revealed that Met deprivation or double deprivation increased cells at G1 phase but decreased those at other phases in the cell cycle (Figure 4B), suggesting that the proliferation of HeLa cells was blocked at the point of entering the S phase under these conditions. There was no increase in aneuploid cells corresponding to dead cells, which confirms the arrest of the cell cycle (Figure 4B). The cell cycle arrest induced by double-free conditions was resumed on supplementation with SAM. To further confirm that the cell cycle was resumed by SAM, we used HeLa/Fucci cells that express Fucci, a cell cycle marker, which permits the dynamics of cell cycle progression in live cells; G1-, G1-/S-, and S-/G2-/M-phase cells to be observed. Consistent with this, double deprivation induced G1-arrest while supplementation with SAM, but not SAH, released the cycle arrest in HeLa/Fucci cells (Figure 4C). These collective results suggest that DNA methylation and the subsequent cell cycle progression appears to be involved in the execution of ferroptosis under conditions of cystine deprivation.

## 4. Discussion

In the current study, we found the Met/cystine double deprivation strongly prevented the execution of ferroptosis under conditions of intracellular Cys/GSH starvation, which led to the survival of HeLa cells as well as Hepa 1–6 cells (Figure 1 and Figure 2, Supplementary Figure S1). Supplementation of SAM, but not its downstream metabolite SAH in Met metabolism, resulted in the increased production of peroxidized lipids and induced ferroptosis in cells under double deprived conditions (Figure 3). On the other hand, SAM supplementation also increased DNA methylation and allowed cell cycle progression to resume (Figure 4). These collective results reveal the pivotal roles of lipid peroxides, the concentrations of which are elevated during cell cycle progression, in ferroptosis execution under Cys starvation conditions.

Cell growth retardation under Met-free conditions is a phenotypic characteristic of some types of cancer cells [12,13,14]. The cell cycle arrest under double-deficient conditions was associated with the suppression of cell death, which indicates that ferroptosis proceeds preferentially in proliferating cells. Observations that the inhibition of xCT triggers ferroptosis in highly proliferating myogenic lines in a MAPK pathway-dependent manner [28] are consistent with the notion that cell growth potentiates the susceptibility to ferroptosis in certain types of cell lines. The findings that the activation of glucose metabolic pathways promotes cell proliferation and simultaneously potentiates ferroptosis via AMPK/mTOR/S6 and MAPK signaling under condition of xCT inhibition [29,30] are also consistent with this notion. 

There appears to be several underlying mechanisms for the cell cycle arrest observed in Met- or double-deficient cell culture. Because Met is an essential amino acid, a defect in Met supply could impair the protein synthesis required for cell cycle progression. The inhibition of protein synthesis during the G2 phase causes a delay in mitotic progression, but may simultaneously preserve Cys for GSH synthesis, thus making cells resistant to oxidative insult [15]. Another possibility is that the cell cycle is regulated by Met metabolites per se. During DNA replication at the S-phase of the cell cycle, methylation at the 5-position of cytosine needs to be maintained in daughter DNA by DNA methyltransferase DNMT1 [16]. A loss of DNA methylation by means of DNMT1 gene ablation causes hemimethylation in the CpG island and leads to cell cycle arrest at the G2 phase but eventual death [17]. Thus, the cell cycle arrest observed in the Met-deficient cultures could, at least partly, be explained by an insufficient supply of SAM from Met metabolism. Consistent with this, SAM supplementation resulted in an elevated DNA methylation and cell cycle progression being resumed (Figure 4).

The origin of the reactive oxygen species (ROS) that triggering lipid peroxidation reactions that execute ferroptosis is under debate. The electron transport chain (ETC) coupled with the tricarboxylic acid (TCA) cycle under Cys starvation conditions appears to be a promising system for explaining this [31,32]. We recently reported that superoxides generated from mitochondrial ETC, notably from complex III, promote ferroptosis under conditions of Cys starvation [25]. ROS production is elevated in response to mitotic stimuli and is involved in various signal transduction processes, which include the activation of growth factor receptor signaling and cell cycle progression mediated by cyclin-dependent kinases, thereby potentiating cell cycle progression [33,34]. ROS production varies at each phase during the cell cycle and peaks in mitosis, resulting in the accumulation of oxidized protein Cys residues in the mitotic phase [35]. As a result, oxidative damage of proteins and nucleotides peaks in mitosis, and when this becomes excessive, could lead to mitotic arrest. Some studies have suggested that ROS that are elevated at specific points of the cell cycle in mitotic progression have a role in this process [36,37]. On the other hand, the intracellular localization of GSH as well as levels of antioxidant enzymes also cyclically changes during the cell cycle. These results suggest that appropriate control of the redox state is essential for cells to successfully progress beyond this checkpoint at the corresponding phase of the cell cycle [38,39].

The restriction in dietary Met has been reported to decrease the concentrations of mitochondrial ETC, and reduce mitochondrial ROS generation in rodents, suggesting a regulatory effect of Met on mitochondrial ETC [40,41]. It has also been reported that dietary Met restriction decreases mitochondrial ROS generation primarily via inhibiting complex I activity and ROS generation rather than augmenting antioxidative capacity, thereby ameliorating oxidative damage to hepatic mitochondrial DNA and proteins [42]. Based on these observations, our findings suggest that metabolic ROS production stimulated by a high metabolic activity increases sensitivity to ferroptosis while Met deprivation causes cell cycle arrest and decreases ROS production, thereby inhibiting the ferroptotic pathway. Future studies should be directed at examining where the ROS come from in executing ferroptosis.

## 5. Concluding Remarks

In summary, we provide evidence to show that metabolic changes as well as cell cycle progression could modulate the cellular sensitivity to ferroptosis. The Met-restriction strategy or ferroptosis-induction strategy could be powerful and potential therapeutic approaches for individual cancer therapy [43]. However as demonstrated in this study, Met restriction may arrest the cell cycle of cancer cells and allow them to escape from ferroptosis, leading to the formation of latent malignant cells. On the other hand, apoptosis-resistant but proliferating cancers may be susceptible to ferroptosis and, hence, a ferroptosis-targeted therapy could be advantageous.

## Data Availability

Not applicable.

## Supplementary Materials

The following supporting information can be downloaded at: https://www.mdpi.com/article/10.3390/cells11101603/s1, Figure S1: Effects of Met/cystine double deprivation on the induction of ferroptosis in Hepa 1-6 cells; Figure S2: Effects of ferrostatin-1 on ferroptosis under Met/cystine double-free conditions but with SAM supplementation.
