# Supplementary material for: Methionine Deprivation Reveals the Pivotal Roles of Cell Cycle Progression in Ferroptosis That Is Induced by Cysteine Starvation
Source: Cells. 2022 May 10;11(10):1603. doi: 10.3390/cells11101603 (PMC9139961; doi:10.3390/cells11101603)
Supplement: Supplementary file 1 [file cells-11-01603-s001.zip › cells-1659908-supplementary.pdf]

Supplementary Figure S1

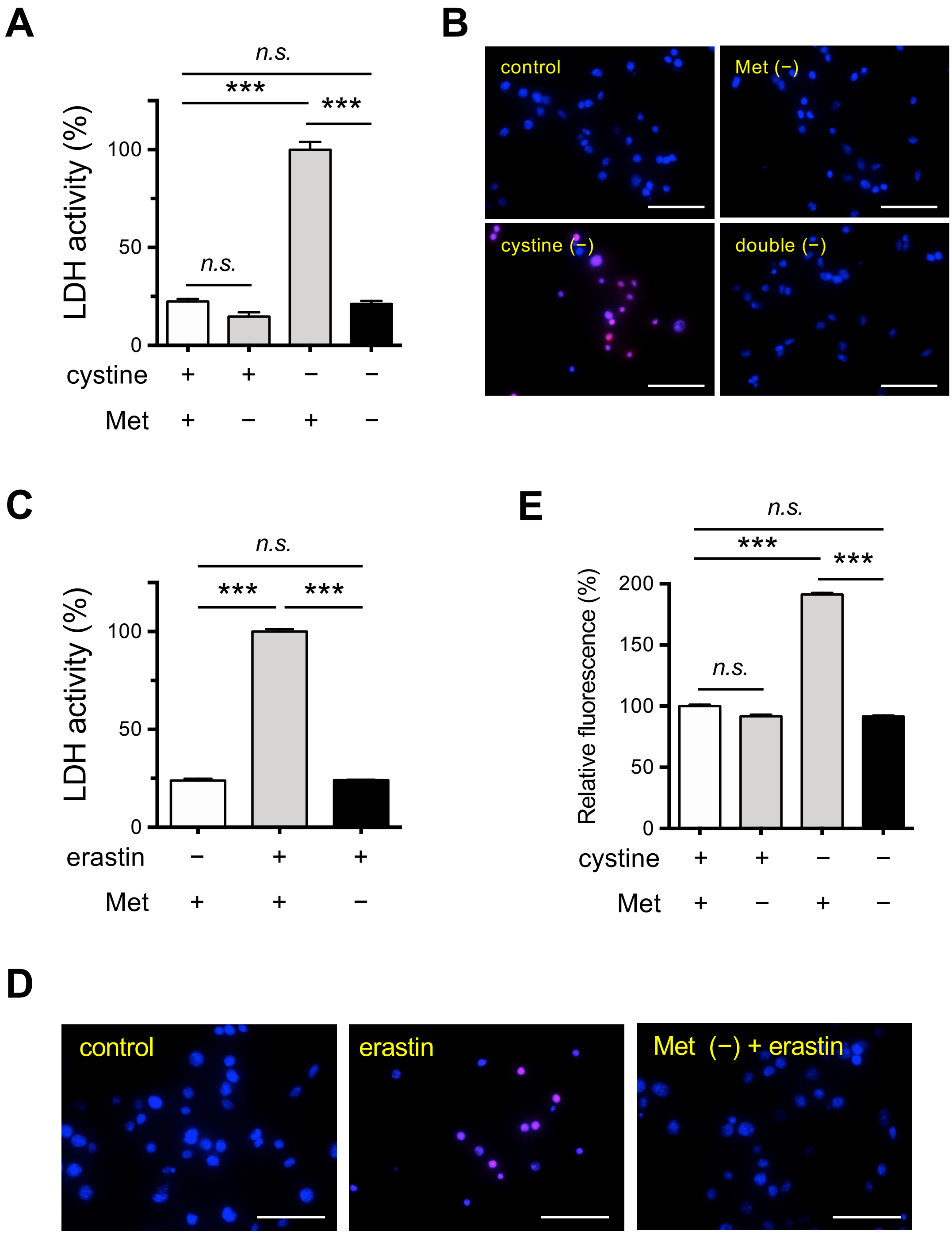

Supplementary Figure S1. Effects of Met/cystine double deprivation on the induction of ferroptosis in Hepa 1-6 cells.

(A) Cytotoxicity of cells assessed by measuring released LDH activity. Hepa 1-6 cells were incubated in complete (control), Met-free, cystine-free, or Met/cystine double-free medium for 24 h. Data represent the mean ± SEM (n = 3). ***$p < 0.001$ (Tukey's test). *n.s.*, not significant.

(B) Plasma membrane integrity of cells that had been treated under the same conditions as (A) was assessed by PI staining. The cells were stained with PI (red) and Hoechst 33342 (blue). Bars: 100 μm.

(C) Cytotoxicity of cells assessed by measuring released LDH activity. Hepa 1-6 cells were incubated in complete (control) or Met-free medium in the presence or absence of 10 μM erastin for 24 h. Data represent the mean ± SEM (n = 3). ***$p < 0.001$ (Tukey's test). *n.s.*, not significant.

(D) Plasma membrane integrity of cells that had been treated under the same conditions as (C) was assessed by PI staining. The cells were stained with PI (red) and Hoechst 33342 (blue). Bars: 100 μm.

(E) Lipid peroxide production assessed by flow cytometry using C11-BODIPY$^{581/591}$. Hepa 1-6 cells were incubated in complete (control), Met-free, cystine-free, or Met/cystine double-free medium for 18 h, treated with 10 μM C11-BODIPY$^{581/591}$, and then subjected to flow cytometry. Values for the fluorescence relative to cells cultured in control medium are shown (n = 3). Data represent the mean ± SEM. ***$p < 0.001$ (Tukey's test). *n.s.*, not significant.

Supplementary Figure S2

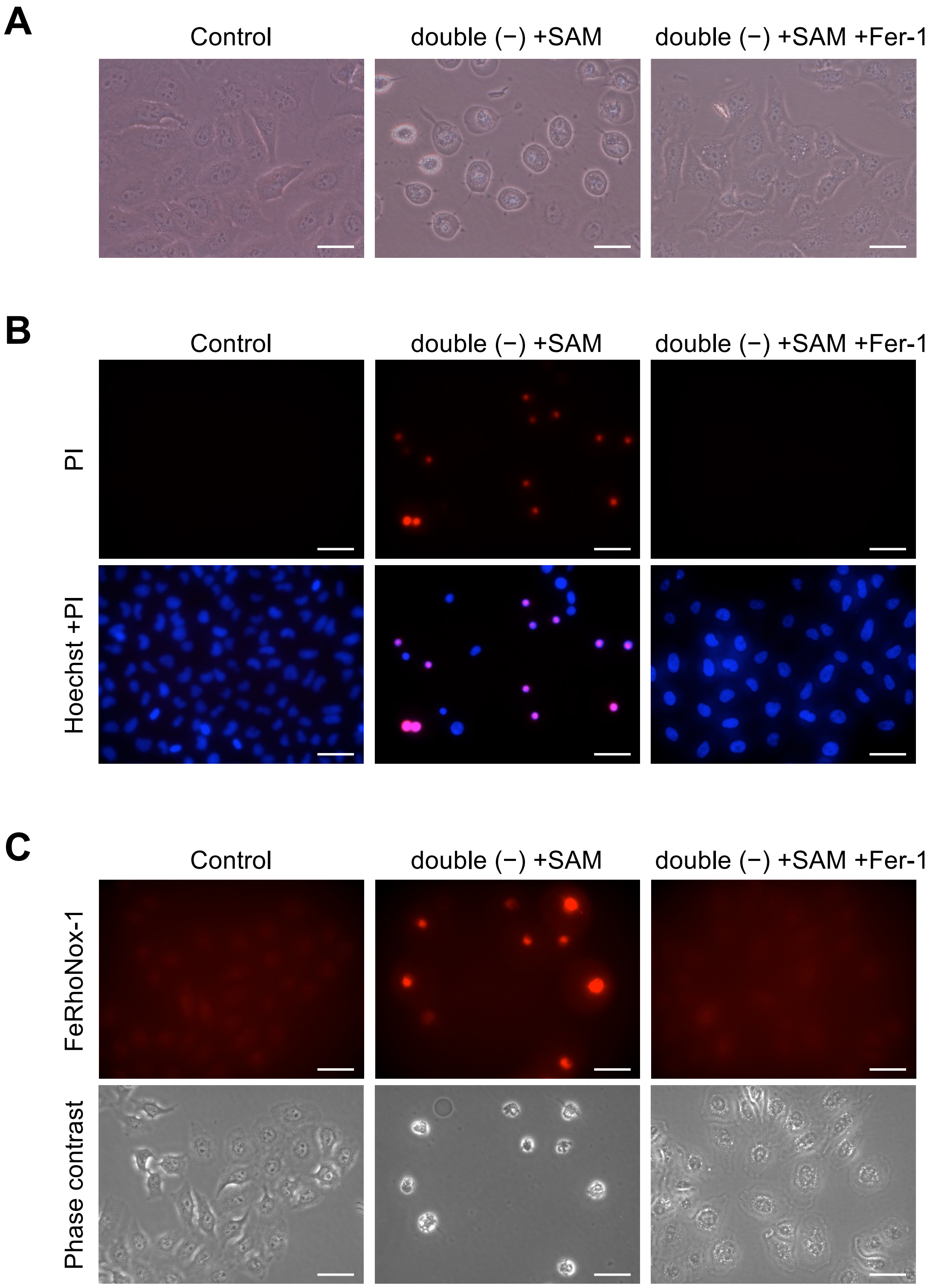

Supplementary Figure S2. Effects of ferrostatin-1 on ferroptosis under Met/cystine double-free conditions but with SAM supplemetation.

(A) Representative phase-contrast images of cells after cystine deprivation. HeLa cells were incubated with complete (control) or Met/cystine double-free medium supplemented with 0.2 mM SAM for 24 h in the presence or absence of 10 μM ferrostatin-1 (Fer-1). Bars: 50 μm.

(B) Plasma membrane integrity of cells that had been treated under the same conditions as (A) was assessed by PI staining. The cells were stained with PI (red) and Hoechst 33342 (blue). Bars: 50 μm.

(C) HeLa cells were incubated with complete (control) or Met/cystine double-free medium supplemented with 0.2 mM SAM for 24 h in the presence or absence of 10 μM ferrostatin-1 (Fer-1), and the intracellular ferrous iron was then visualized. Bars: 50 μm.
